# Editorial: Use of smartphone applications to increase physical activity and fitness, volume II

**DOI:** 10.3389/fpubh.2023.1190566

**Published:** 2023-04-18

**Authors:** Victor Zuniga Dourado, Jayoung Kim

**Affiliations:** ^1^Department of Human Movement Sciences, Federal University of São Paulo (UNIFESP), Santos, SP, Brazil; ^2^Lown Scholars in Cardiovascular Health Program, Harvard T.H. Chan School of Public Health, Boston, MA, United States; ^3^Department of Surgery, Cedars Sinai Medical Center, Los Angeles, CA, United States; ^4^Department of Biomedical Sciences, Cedars Sinai Medical Center, Los Angeles, CA, United States

**Keywords:** physical activity, exercise, gamification, fitness applications, health applications

The benefits of physical activity to prevent non-communicable diseases and several types of cancer are evident in Bull et al. (1). Despite its unquestionable health benefits, one in four (27.5%) adults (2) and more than three-quarters (81%) of adolescents (3) do not meet the recommendations for aerobic exercise (1). These data also reveal no overall improvement in global levels of participation over the last two decades (1). The current guidelines are “every step count,” i.e., “doing some physical activity is better than doing none.” In this context, technology can play an essential role in breaking sedentary behavior. Recent technological advances such as smartphones and their apps have been released to interact with and evaluate the human bodily activity and current literature supports the use of wearable and mobile equipment to increase the level of physical activity in adults (4–6). The effect of such interventions can be considered small but significant, with strategies such as goal setting, feedback on behavior, self-monitoring, and social support highlighted as most promising (7, 8). In this special Frontiers in Public Health issue, five articles explored important aspects of using technology to increase adult physical activity and fitness.

Liu et al. evaluated the mediating role of the e-platforms physical activity among the Chinese people during the COVID-19 lockdown. Preventive measures related to COVID-19 included washing hands, keeping social distance, wearing masks, staying at home, darning lockdown, quarantine, doing physical activity while staying at home, and monitoring the health were determinants for the level of physical activity of the participants. The use of Fitness Apps, Live Streaming Workout Classes, and Virtual Reality Fitness showed significant indirect effects on physical activity, i.e., positive mediating roles. Thus, smart applications may play an essential role as an alternative to gyms and change people's perspectives regarding the adoption of health and fitness.

To help patients monitor their health in real-time, Li and You proposed an intelligent mobile health monitoring system (Im-HMS) and establishes a corresponding health network to track and process patients' physical activity and other health-related factors in real time. The experimental results show that, in general, the Im-HMS system proposed in this study is more accurate than the user-centered decoding (UCD) system and has a lower delay, lower error, and higher efficiency, and energy utilization is more efficient than the UCD system, which is of great importance for mobile health monitoring in practical applications.

Unfortunately, the effects of using technologies to increase the physical activity level of adults have been wrongly investigated and only in the short term. Aiming to investigate the long-term effects of digital interventions on the physical activity level of adults, Zanaboni et al. propose a hybrid Type I effectiveness-implementation randomized controlled trial (ONWARDS study) (9) targeting an inactive and presumably high-risk population. The hypothesis is that the combination of effects of an activity tracker with the personalized metric Personal Activity Intelligence (PAI), access to online training videos (Les Mills+) to perform home-based training, and additional peer support *via* social media will be most effective in increasing participants' physical activity level after 18 months. Multicomponent interventions appear to be significantly more effective than those based only on the application. The best combination of features, behavior change techniques and the level of contact necessary with the user to maximize engagement remain to be determined in the literature (10).

However, the dynamic context of smartphone apps, for example, demands adaptive interventions. Therefore, the efficiency of conventional clinical trials is questionable. The Multiphase Optimization Strategy (MOST) could be used in the post-pandemic to identify the combinations and intensities of strategies to change behavior in favor of physical activity (11). First, clinical trials with mricorandomizations for immediate interventions are the greatest advantage of technological interventions and have been very little used (12). They can identify the best multicomponent strategies to increase physical activity proximally and distally (13). Then, although feasible and applicable, the Sequential Multiple Assignment Randomized Trial (SMART) designs, which allow re-randomizations throughout the intervention, should be used to tune the intensity of interventions previously defined in the micro-randomizations. This strategy has already proven to be feasible (14) and has already been suitable for use to increase the level of physical activity in adults (15). Therefore, the use of adequate designs to investigate the effects of smartphone apps on physical activity is fundamental when thinking about greater adherence. For this type of technology to make a difference inside or outside the context of the pandemic, the applications must be developed on a scientific basis (16).

In this Research Topic Amer et al. showed that the proportion of individuals using mHealth apps is high (47.9%), with the most common apps being the ones related to daily steps-counting (54.2%), indicating the great potential of mobile technologies to encourage healthy behaviors. The main advantages of using mHealth apps were saving time (64%), the possibility of following up on the health status at any time (48.9%), and getting correct information (40.9%). The main reported disadvantages included not studying the medical situation thoroughly (63.1%), a lack of continuous follow-up from a specialist (52.9%), and a low quality of diagnosis and follow-up (40.6%). Sharing health files or laboratory results with the follow-up specialists (73.5%), linking the health information of the users to their health files (63.3%), following up on health status using charts (56.3%), and adding health information about specific diseases (54.1%) could improve the usage of the mHealth apps. On the other hand, unfortunately, this technology has an important barrier to overcome. The vast majority of mHealth application users are highly educated, have a higher socioeconomic level and formal employment, and are younger. The profile of smartphone app users for physical activity in developing countries is despicably known. Accordingly, the study by Vieira et al. confirmed that smartphone applications in this context, also in a developing country, users of APP were younger and had higher education, lower cardiovascular risk, better socioeconomic status, a better quality of life, better cardiorespiratory function, better body composition, greater physical fitness and more moderate to vigorous physical activity in daily life. The results of the multiple logistic regression showed that age, arterial hypertension, cardiorespiratory fitness, socioeconomic status, and quality of life were the variables most significantly associated with the use of the apps.

Therefore, there is great potential for mobile technologies to increase the level of physical activity in adults. However, the effects of these technologies must be better evaluated in appropriate designs, their development must be science-based and should consider socioeconomic and cultural differences, and, most importantly, must be directed toward those who can benefit most from it, e.g., people with lower levels of physical activity, lower cardiorespiratory fitness, and a higher cardiovascular and metabolic risk. Undoubtedly, this will be the main challenge for future studies in this area of knowledge.

## Author contributions

VD contributed to the writing of this editorial. VD and JK contributed to the article and approved the submitted version.
